# Considerations for Co-creating a Culture of Well-being at your Medical Institution

**DOI:** 10.1007/s40670-025-02559-6

**Published:** 2025-12-05

**Authors:** Delaney Lockwood, Amy Baldwin, Casey N. Bassett, Janette R. Hill, Cathy Snapp

**Affiliations:** 1 AU/UGA Medical Partnership, Medical College of Georgia, Athens, GA 30602 USA; 2 Department of Interdisciplinary Biomedical Sciences, Humanism in Medicine, Office of Personalized Health and Well-Being, University of Georgia School of Medicine, Medical College of Georgia, Russell Hall, Room 235Q 1425 Prince Avenue, Athens, GA 30602 USA

**Keywords:** Undergraduate Medical Education, Resilience, Well-being

## Abstract

Medical students face tremendous challenges during their medical training. To better support our medical learners, we should create institutional culture that promotes well-being and provides students with tools needed to prioritize their mental, emotional, and physical health. This guide offers considerations for co-creating and implementing a culture of well-being at medical institutions with an emphasis on team building, leadership, engagement, adaptability, and sustainability. When learners are supported in an institution that values well-being, positive impacts include improved performance and resilience, increasing the likelihood of training medical professionals who are equipped to provide humanistic healthcare.

## Introduction

Medical learners and physicians experience a high rate of burnout and other mental health challenges, which in turn impacts self and patient care [1]. Peers can play a powerful role in helping fellow learners overcome these challenges. Using a peer-led, faculty supported model, a culture of well-being can be cultivated at medical institutions to promote resilience and overall well-being in medical learners [2]. A collaborative approach that includes all stakeholders (administrators, faculty, staff, learners) working towards a common goal of increasing resilience and well-being allows for robust, creative, and adaptive solutions. Co-creation of well-being programming encourages buy-in, input, ownership and skill development that resonates individually and impacts institutional culture.

**Fig. 1:**
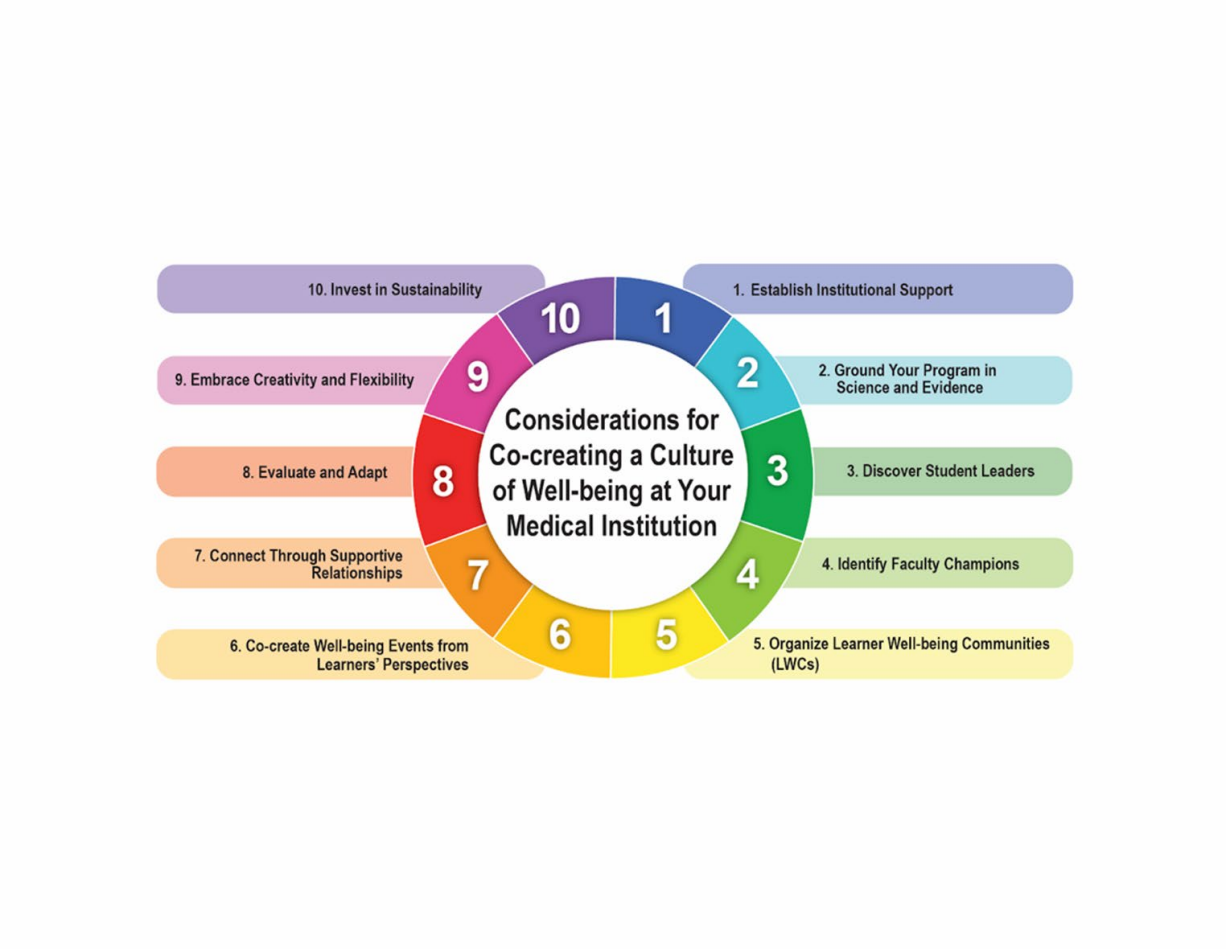
Considerations for Co-creating a Culture of Well-being at Your Medical Institution

Personalized resilience is a comprehensive approach to well-being that incorporates several strategies, tools, and techniques to build positive emotion and promote sustainable growth, *in the moment of need*. Personalized well-being is grounded in a strength-based approach to emotional resilience. It addresses the positive emotional states and inner neural traits that promote system-wide balance, bringing together critical components such as body awareness, positive lifestyle choices, and emotional regulation techniques to reduce stress and promote well-being [3]. A personalized approach to well-being is uniquely designed to meet learners “where they are” and help identify innate, core resources available to them, to broaden and build an array of skills and techniques for optimized emotional regulation, performance and health promotion [4]. Figure 1 provides considerations to help guide those wanting to co-create a culture of well-being at their institution, from establishing institutional support to organizing learner well-being communities to embracing creativity and flexibility. Each consideration is discussed in more detail in the following sections.

This figure shows 10 considerations for co-creating a culture of well-being, with its cyclical nature reinforcing the importance of the co-creation process that occurs each academic year.

### Establish Institutional Support

In establishing a culture of well-being at academic medical institutions, a foundational step is garnering support from key leaders and administrators. For example, a Dean’s endorsement of an institution-wide well-being program shows support for creating opportunities to incorporate flourishing in medicine as a focus and priority for medical learners, faculty, staff, and administration. Institutional support is key for co-creating a culture of well-being and promoting buy-in, which fosters a collaborative effort where all stakeholders are invested in the success of resilience efforts.

Emphasis on well-being during orientation from Day 1 of a medical learner’s experience shows a commitment to and prioritization of overall health and wellness. Well-being events should be planned in advance and incorporated throughout the academic year with institutional support at all levels. This support should also include a robust budget to promote the creation and implementation of well-being programming.

There may be instances where well-being programming is being developed by faculty in order to garner institutional/leadership level support. Established and recognized models for well-being initiatives, along with learner feedback, can assist with establishing a robust well-being program. For example, Kern’s 6-step model, American Medical Association’s (AMA) 8-step toolkit, as well as learner feedback from the Graduate Questionnaire (GQ) can all be leveraged to assist with development, as well as demonstrate the need for the programing to leadership [5, 6]. The establishment of administrative support for flourishing in medicine ensures scheduling, funding, and the sustainability of programming.

### Ground your Program in Science and Evidence

Personalized resilience efforts are grounded in attaining competency in educational milestones, which form an evidence-based foundation of resilience for medical learners and faculty. Implementing programming initiatives anchored in the emerging science of emotional self-regulation, positive neuroplasticity, sustainable habit change, health promotion, and a growth mindset results in a comprehensive training model that is both richly personalized and prized for its in the moment, on demand use and efficacy [7, 8]. It is essential to provide evidence-based practices and supporting research for learners’ foundational knowledge. Providing knowledge and experiential learning, through the personalized application of research-based skills, medical learners and their faculty report higher levels of ‘buy-in’ and application of well-being tools, generating greater health promotion in the learning environment.

[3].

There can be numerous challenges commonly encountered in seeking to build buy-in and support for well-being initiatives at the institutional level such as: budget constraints, lack of identified governance at the leadership level, and well-being viewed as non-core/para-curricular. Several successful strategies include (1) seeking seed grant funding to identify promising well-being programs, (2) selecting an identified Director or Dean at the administrative level to take responsibility for the development and implementation of a comprehensive, integrated well-being institutional strategy and (3) normalizing the importance, value and utility of well-being skills and lifestyle habits through building a strong network of dedicated faculty champions who serve as supportive mentors and inspiring role models [9].

This training-based focus on human growth and flourishing, grounded in positive neuroplasticity and tied to high yield impact on performance and sustained emotional regulation, is crucial in competitive, academic environments both nationally and globally [10]. Internationally, systemic approaches to well-being differ by culture and region but several common core implementation practices can be identified. There appears to be universal agreement that resilience models are more effective when implemented through a top-down approach, i.e. well-being mandates at the executive leadership level. Second, there is a global trend for the initiation of national accrediting standards and culturally appropriate well-being frameworks within academic medicine environments. Lastly, well-being content and experiential learning embedded within the curriculum through medical humanities, professionalism, ethics and cultural competency is prioritized.

### Discover Student Leaders

Through intrinsic motivation and passion for creating a culture of well-being at their institution, students have the opportunity to further develop leadership skills in their areas of interest. When planning a well-being initiative at your academic medical institution, it is vital to collaborate with student leaders. Students who are dedicated leaders co-create and drive well-being programming with the support of faculty and staff. Depending on the size of your institution, a small set of 3–10 student leaders can make a large impact on cultivating a culture of well-being. Recruit student leaders who (1) place a high value on, (2) have an established understanding of, and (3) practice well-being in their own lives. These leaders may be identified as learners who regularly attend well-being sessions or who are involved in student interest groups where well-being is a focus. Peer leaders can be recruited via email or group messaging in addition to word-of-mouth connections amongst students and faculty. It is important to take an inclusive approach when recruiting student leaders, as differing perspectives contribute to more culturally sensitive leaders who work to provide well-being opportunities and modalities that are relatable to the entire cohort.

After identifying student leaders, roles should be established. Roles may vary and could include: president or head student leader, event planner, communication director, research lead, treasurer, secretary, and community outreach executive. These leaders can regularly meet to brainstorm event ideas, relay needs of the student body to faculty and administration, peer coach other students in emotional resilience training, and lead well-being events.

They can also empower and inspire their classmates to get involved in well-being activities so that the impact of these programs can be felt across student cohorts. Students should have the autonomy to make decisions and adaptations for well-being programming with the unwavering support from faculty, staff, and administration.

### Identify Faculty Champions

Faculty champions prioritize and openly support learner well-being by demonstrating a holistic and learner-centric approach to teaching, advising, coaching and mentoring students. They recognize the importance of learner resilience and its connection to academic and professional success. Some faculty champions self-identify, while others can be directly invited to participate by administration before the start of each academic year. In an academic medicine setting, faculty can take advantage of continuous skill development opportunities in resilience and well-being. For settings where this faculty development is not offered on-site institutionally, there are many training and certificate programs that are offered both in-person and virtually. Institutional support for faculty time and training is crucial for the success of well-being programming and the improvement of institutional culture.

Faculty champions are embedded within the institutional community to support, guide, coach and mentor learners. They are active participants in well-being communities and events and help promote and encourage learner involvement. Faculty support of students positively impacts the institutional culture of well-being and overall well-being of all stakeholders.

### Organize Learner Well-being Communities (LWCs)

The success of learner well-being communities (LWCs) lies in the strength of their composition. LWCs should include learners, peer coaches (student leaders), and faculty champions. LWCs should be small enough to promote a better sense of belonging and connection, allowing the formation of closer relationships of support, especially at academic medical institutions with larger-sized cohorts. Members of each new incoming class should be assigned to a LWC assembled on the first day of orientation, ideally keeping community sizes to less than 20 new students each. This allows interaction, bonding, and support throughout the first week of classes. Each LWC should also have designated 1–2 peer coaches and 1–2 faculty champions.

Communities create a quicker sense of belonging and connection among first-year students, and interactions with their well-being peer coaches and faculty champions provide immediate mentors and supporters to assist in their transition into medical school. To build camaraderie, each LWC can be asked to develop a supportive team motto and cheer. Each community is encouraged to earn points through their organization of and participation in well-being activities throughout the year, with a LWC winner declared at the end of the academic year. Continuity of community names each year allows for each cohort of students to continue participating and feel connected to their designated community throughout all four years of their undergraduate medical education (UME) [11]. Impact of the LWCs can be evaluated through participation as well as additional data collection efforts (e.g., surveys, focus groups) from members.

### Co-create Well-being Events from Learners’ Perspectives

Prioritizing and embedding well-being activities into the heart and soul of medical education is valued by medical learners [11; unpublished data]. Students have a wide variety of interests and definitions of “well-being.” Not all students will respond to traditional well-being events that involve activities such as meditation or yoga. Students should be polled through surveys and small focus groups to determine what events they would like to create and/or participate in each year. As each learner cohort may vary in their preferences, every class should be consulted annually.

After determining ideas and class preferences for well-being events, peer leaders can work together to clarify event preferences for the academic year. Coordination with faculty, staff and administration prior to and throughout the year can ensure feasibility of activities and events, including financing and scheduling. Providing a mechanism for on-going idea sharing from all stakeholders (e.g., quick email survey, inviting ideas through leadership lunch meetings) reinforces a collaborative approach. The role of the faculty, staff and administration should be one of empowerment, helping to enable and support student ideas and efforts allowing them to maintain buy-in and ownership of their own well-being programming.

Peer leaders and faculty can coordinate events, create event advertisements, and secure materials needed. Providing myriad activities, robustly publicized and scheduled around students’ preferred time slots ensures a wide array of supportive, health-promoting events and sessions for medical learners and their faculty champions. Utilizing peer leaders to promote events is a highly effective mechanism for successfully implementing well-being programming [12]. One to two large events per semester, along with weekly to monthly smaller events such as brief resilience-building sessions, are typically feasible.

Examples of well-being events include cooking together, painting or pottery sessions, hiking, breathing exercises, community service events, or even “Make Your Own Smoothie” days. Medical improv is another example of a skill-building, well-being event [13, 14]. Improv games not only help individuals reflect, they are a fun way to explore status, practice teamwork, narrative building, and mindfulness. Offering other medical humanities activities (e.g., dance, music) extend possibilities and highlight the Fundamental Role of Arts and Humanities in Medicine [15]. Demonstrations by integrative medical practitioners (e.g., Reiki, acupuncture) can also provide a way to explore complementary approaches in medicine while providing events where learners can connect with community practitioners and each other.

In addition to events that are hosted throughout the academic year, students should be offered training programs focused on building skills in emotional resilience and overall well-being. Some students may prefer personalized one-on-one coaching sessions tailored to their individual needs, while others may enjoy group events to learn while bonding and connecting with others through shared experiences. Peer leaders for each well-being group can lead these activities in addition to faculty champions.

### Connect Through Supportive Relationships

Support from varied perspectives and roles is a key consideration for well-being programs. Mentoring is an important type of support and can range from formal to informal mentoring amongst peers and faculty [16]. Determining which mentoring and coaching activities should be included in a well-being program is important to consider. Tools such as the Mentoring Wheel can be useful for providing a frame for mentoring activities as a part of larger well-being programming [17].

Peer leaders are fundamental to the coaching process as well as providing leadership for well-being programming. Peer learning provides a foundation for guiding and establishing a formal peer support framework [18]. Providing coaching training opportunities to the peer leaders can assist with overall effectiveness. Additionally, the importance of confidentiality and the practice of safe boundaries are part of the synchronous training experiences medical students receive. There are several peer coaching models that can be used to facilitate the training [3].

Faculty champions are also important as mentors for learner well-being. By pairing faculty champions with peer coaches in a team, mentoring opportunities are enabled at multiple levels. Working collaboratively with peer coaches, faculty champions can offer support for the team of medical learners as well as co-facilitate planned activities. Faculty champions can also support medical learners through one-on-one interactions such as individualized resilience coaching sessions. Importantly, faculty champions also provide a bridge to the larger institutional commitment to a culture of well-being.

### Evaluate and Adapt

Evaluation of well-being programming is key to guiding, planning, conveying commitment to, and ensuring continuous quality improvement (CQI). Gathering data in a variety of ways (e.g., surveys, interviews) and from multiple stakeholders (e.g., learners, faculty champions, staff, administrators) is important to the evaluation process. At the program level, pre- and post-well-being session surveys and post-session interviews can provide a basis for making evidence-based decisions on the design, development, evaluation, revision and implementation. Additional data from student end-of-year surveys can also be used to inform adaptation for future well-being programming. Continuing to gather data across cohorts can be used to evaluate cultural changes over time.

On an institutional level, data from the AAMC Medical School Graduate Questionnaire (GQ) and the Liaison Committee for Medical Education (LCME) Independent Student Analysis (ISA) can be gathered to gain insight into overall perceptions of well-being [19, 20]. The expanded lens of these data sources can assist with gaining insights into more specific programming possibilities. Since incorporating our co-creation and collaborative approach with learners, our program’s well-being data from the GQ and ISAs have improved significantly, demonstrating the effectiveness of our continual co-creation and adaptation of well-being programming. The GQ and ISA data can also be used to inform potential collaborative scholarship activities amongst learners and faculty. Institutional level data can provide a robust source of information for idea generation and decision making.

### Embrace Creativity and Flexibility

Well-being programming provides the opportunity for embracing creativity and flexibility during the planning and implementation processes. Learners, faculty, administration, and staff all bring creative ways to facilitate connection across various learner cultures. Each new cohort of peer leaders and faculty champions may have new ideas and plans that should be welcomed with enthusiasm and motivation. When stakeholders have representation in the co-creation of well-being events and activities, there is a greater chance that sessions will resonate with individuals and promote connection.

Co-creating a variety of activities and events that facilitate connection and serve diverse biopsychosocial needs are vital for fostering overall well-being [21]. Flexibility and creativity are needed for both the content and scheduling of events. Consulting the academic calendar for important exam and testing dates, as well as checking in with learners about their scheduling preferences, are crucial for planning well-being events. Activities planned before and after “high stress” events have higher success rates and may also result in higher participation [22]. By providing a variety of well-being programming throughout the year that has been informed by learner preferences and carefully scheduled, learners have the flexibility to attend events and sessions that resonate most with them.

Lastly, it is important to embrace flexibility and creativity when adapting well-being programming based on evaluations. Feedback throughout the academic year can inform changes that can be made to enhance the program’s impact on present and future cohorts. Alterations in strategies and approaches may need to occur during each academic year in order to better serve the present cohort of medical learners. As part of a continuous quality improvement process, it is also important to highlight how well-being programming is influenced and modified as a result of feedback obtained. Learners appreciate contributing to the growth and evolution of their own development as well as improving institutional culture. This feedback loop ensures that contributions are valued and incorporated when appropriate, adding yet another level to the buy-in and ownership of everyone’s responsibility to co-create an institutional culture of well-being and resilience. Student feedback should be approached with an open-mind and dedication to continual improvement. This flexibility and creativity in problem-solving embodies the essence of co-creation and fosters an ever-evolving culture of well-being for medical learners.

### Invest in Sustainability

Sustainability is a component of a well-being program that should be approached with continuous and dedicated support from faculty, staff, and administration [23]. This is essential, as well-being programming requires consistent flexibility. Co-creation with and adaptation to the needs of learners is key to sustaining programming that is relatable and effective. For example, our peer coaching model is now in its fifth year, and with each new academic year, we have used feedback and guidance from previous participants to expand and improve upon the training needs of our peer coaches. The adaptability and sustainability of this peer coaching model is evidenced by an increase in the number of medical learners who volunteer to undergo training and become peer coaches each year. Establishing a program framework, including using team names and defining a process for identifying new student leadership each academic year, provides needed program consistency. However, to be truly sustainable, administration and faculty leadership must adapt to each new cohort of medical learners and their well-being needs. Consistent support, flexibility, and adaptation each year are all key to sustaining a relevant and impactful well-being program.

## Conclusion

Sustained stress and other lifestyle challenges lead to a reduction in the healthcare workforce due to decreased mental clarity, fatigue, lack of work/life balance, health challenges, and lost productivity. Many of today’s medical educators and learners have not had the opportunity to learn how to use well-being strategies and resilience tools as powerful change agents for health promotion and flourishing in medicine. Therefore, this article aims to provide considerations for implementing programming at medical institutions that help cultivate a culture of well-being and provide strategies for creating personalized well-being plans. By co-creating well-being programming that is peer-led and supported by faculty, staff, and administration, medical learners can thrive both academically and personally. Medical learners can apply emotional resilience skills beyond their training into clinical practice to ultimately provide holistic, humanistic patient-centered care. Ultimately, investing in the well-being of medical learners today creates a ripple effect that extends to the broader healthcare system and the communities they serve, contributing to a healthier, more sustainable public health landscape for generations to come.

## Data Availability

N/A.
